# Selective brain hypothermia attenuates focal cerebral ischemic injury and improves long‐term neurological outcome in aged female mice

**DOI:** 10.1111/cns.14017

**Published:** 2022-11-07

**Authors:** Liqiang Liu, Jia Liu, Ming Li, Junxuan Lyu, Wei Su, Shejun Feng, Xunming Ji

**Affiliations:** ^1^ Beijing Institute of Brain Disorders, Laboratory of Brain Disorders, Ministry of Science and Technology, Collaborative Innovation Center for Brain Disorders Capital Medical University Beijing China; ^2^ Beijing Institute of Geriatrics, Xuanwu Hospital Capital Medical University Beijing China; ^3^ Department of Neurology, Xuanwu Hospital Capital Medical University Beijing China; ^4^ Department of Neurosurgery, Beijing Tsing Hua Chang Gung Hospital, School of Clinical Medicine Tsing Hua University Beijing China; ^5^ Department of Neurosurgery, Xuanwu Hospital Capital Medical University Beijing China

**Keywords:** aged mice, female, hypothermia, ischemic stroke, protection

## Abstract

**Aims:**

This study aimed to investigate the effects of mild selective brain hypothermia on aged female ischemic mice.

**Methods:**

A distal middle cerebral artery occlusion (dMCAO) model was established in aged female mice, who were then subjected to mild selective brain hypothermia immediately after the dMCAO procedure. Neurological behavioral examinations were conducted prior to and up to 35 days post‐ischemia. Infarct volume, brain atrophy, pro‐inflammation, and anti‐inflammation microglia/macrophages phenotype and white matter injury were evaluated by immunofluorescence staining. Correlations between neurological behaviors and histological parameters were evaluated by Pearson product linear regression analysis.

**Results:**

Sensorimotor and cognitive function tests confirmed the protective effect of mild selective brain hypothermia in elderly female ischemic mice. In addition, hypothermia decreased the infarct volume and brain atrophy induced by focal cerebral ischemia. Furthermore, hypothermia alleviated ischemia‐induced short‐term and long‐term white matter injury, which was correlated with behavioral deficits. Finally, hypothermia suppressed the harmful immunological response by promoting the transformation of pro‐inflammatory microglia/macrophages to anti‐inflammatory phenotype. This polarization was negatively correlated with neuronal loss and white matter injury.

**Conclusion:**

Mild selective brain hypothermia promoted long‐term functional recovery by alleviating white matter damage in an aged female mouse model of ischemia.

## Graphical abstract

Selective brain hypothermia alleviated ischemia‐induced neuronal loss and white matter injury, which was correlated with behavioral deficits. Furthermore, selective brain hypothermia suppressed the harmful immunological response by promoting the transformation of microglia/macrophages from the M1 to M2 phenotype. This polarization was negatively correlated with neuronal loss and white matter injury.

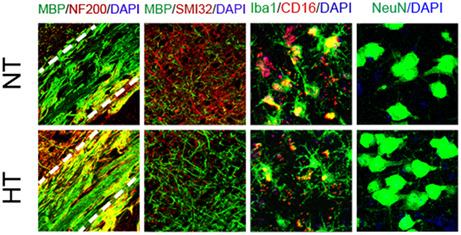

## 1 INTRODUCTION

Ischemic stroke is the most common cerebrovascular disease with high morbidity, disability, and mortality, especially in developing countries.^1^ To date, interventions for ischemic stroke are limited, and various clinical translational trials of preclinical neuroprotective strategies have failed.^2^, ^3^ Although the precise reasons for clinical translational failure remain unknown, one reason may be related to the fact that the majority of preclinical studies focus only on male adults. Clinically, ischemic stroke occurs predominantly in the elderly, in both males and females.^4^ Interestingly, there are differences in the severity, outcome, and response to different therapeutic strategies for ischemic stroke between aged males and females.^5^, ^6^ Preclinical research has also found that some neuroprotective strategies work only in male but not female animals in models of ischemic stroke.^7^, ^8^ Therefore, in terms of age and gender, improving the matching degree between preclinical and clinical trials may be an important way to improve the success rate of clinical trials and promote the clinical translation of stroke intervention strategies.

Hypothermia is a robust neuroprotective strategy that has been applied in experimental ischemic stroke.^9^, ^10^, ^11^, ^12^, ^13^ We previously found that mild selective brain hypothermia played a neuroprotective role in an ischemia model in adult male mice.^10^ However, since the lack of consideration of age and gender in our and other previous studies may limit the clinical translation of hypothermic therapy, it is imperative that we validate the efficacy of therapeutic hypothermia in female and elderly ischemia models to promote clinical translation.

In this study, we induced focal brain ischemic injury by permanent distal middle cerebral artery occlusion (dMCAO) in aged female mice and then placed a simple cooling device on the heads of the mice to investigate the role of mild selective brain hypothermia. We found that selective brain cooling improved the neurological outcome, reduced infarct volume and brain atrophy, protected gray and white matter, and promoted polarization of microglia/macrophages toward an anti‐inflammatory phenotype. These results showed that selective brain hypothermia has neuroprotective effects in an ischemic stroke model in aged female mice.

## 2 MATERIALS AND METHODS

### 2.1 Animal model

Female, aged (18 months old) C57BL/6 mice (Charles River Laboratories) were used in our study. All experimental procedures were approved by the Animal Care and Use Committee at Capital Medical University. Mice were housed in a humidity‐ and temperature‐controlled pathogen‐free facility with a 12‐h light/dark cycle. By using a lottery drawing box, animals were randomly assigned to normothermia (NT), hypothermia (HT), or sham (S) groups. Permanent dMCAO was performed in the left hemisphere by occluding the left common carotid artery and left distal middle cerebral artery as previously described.^14^ Regional cerebral blood flow (rCBF) was measured by Laser Doppler flowmetry. Mice whose rCBF was not reduced to <30% of their pre‐surgery baseline were excluded. All experimental outcome data were collected by investigators blinded to the experimental designs. Sham mice underwent the same surgical procedures, but did not undergo occlusion of the common carotid artery and distal cerebral middle artery.

### 2.2 Administration of hypothermia

Selective brain cooling was induced immediately after the dMCAO procedure had been completed. The cooling procedure was carried out as described in our previous study.^11^ Briefly, a cooling pad was placed over the mice skulls, and the sub‐temporalis muscle temperature was monitored and maintained at 31–32°C. After 50 min cooling, mice were allowed to rewarm passively to normal temperature. NT and S group mice were anesthetized for 50 min but maintained normal rectal temperature using a heating pad (Physitemp; TCAT‐2LV controller).

### 2.3 Neurological behavioral tests

Neurological behavioral examinations were conducted prior to and up to 35 days post‐ischemia as previously described.^15^ Rotarod and adhesive removal tests were performed to evaluate the sensorimotor functions before ischemia and 3, 5, 7, 10, 14, 21, 28, and 35 days after ischemia. Morris water maze^16^ and passive avoidance^17^ tests were used to evaluate the long‐term cognitive deficits at 22, 23, 24, 25, 26, 27, 34, and 35 days post‐dMCAO. All neurological function tests were performed by investigators blinded to the experimental designs.

### 2.4 Immunofluorescence staining

Mice were sacrificed 7 and 35 days post‐dMCAO. Coronal slices (25‐μm‐thick) were prepared for immunofluorescence staining as previously described.^18^ The following primary antibodies were used: rabbit anti‐NeuN (EMD Millipore), rabbit anti‐microtubule‐associated protein 2 (MAP2; sc‐20,172; Santa Cruz), mouse anti‐200kD neurofilament heavy (NF200; MAB5262; Millipore Sigma), rabbit anti‐beta‐amyloid precursor protein (β‐APP; 512700; Fisher Scientific), rabbit anti‐myelin basic protein (MBP; ab5622; Abcam), goat anti‐Iba1 (ab5076; Abcam), goat anti‐CD206 (AF2535; R&D Systems), rat anti‐CD16 (553142; BD Pharmingen), and mouse anti‐SMI‐32 (ab50761; Abcam). Samples were blocked in 5% normal donkey serum (Jackson ImmunoResearch) to reduce nonspecific binding. When a mouse primary antibody was used, a mouse‐on‐mouse (M.O.M) blocking reagent (MKB‐2213; Vector Laboratories) was used to avoid cross‐reactivity. Donkey Cy3‐ or DyLight 488‐conjugated secondary antibodies (Jackson ImmunoResearch) were used. Images were obtained using an Olympus FluoView FV1000 confocal microscope (Olympus America) and analyzed with ImageJ. Cell numbers were calculated from two random microscopic fields in each mouse brain.

### 2.5 Statistical analysis

All statistical analyses were performed by investigators blinded to the experimental design. Data were expressed as mean ± standard deviation (SD). Variables were checked for normality using the D'Agostino & Pearson test or Shapiro–Wilk normality test. For two group normal distribution and equal variances data, the unpaired *t*‐test (two‐tailed) was performed. Otherwise, nonparametric tests were used. For multiple groups, one‐ or two‐way analysis of variance (ANOVA) followed by the Tukey post hoc test was carried out. Correlations between neurological behaviors and histological parameters were evaluated by Pearson's product linear regression analysis. A *p* value <0.05 was deemed as statistically significant.

## 3 RESULTS

### 3.1 Mild selective brain hypothermia alleviates long‐term neurological deficits induced by acute cerebral ischemia in aged female mice

**FIGURE 1:**
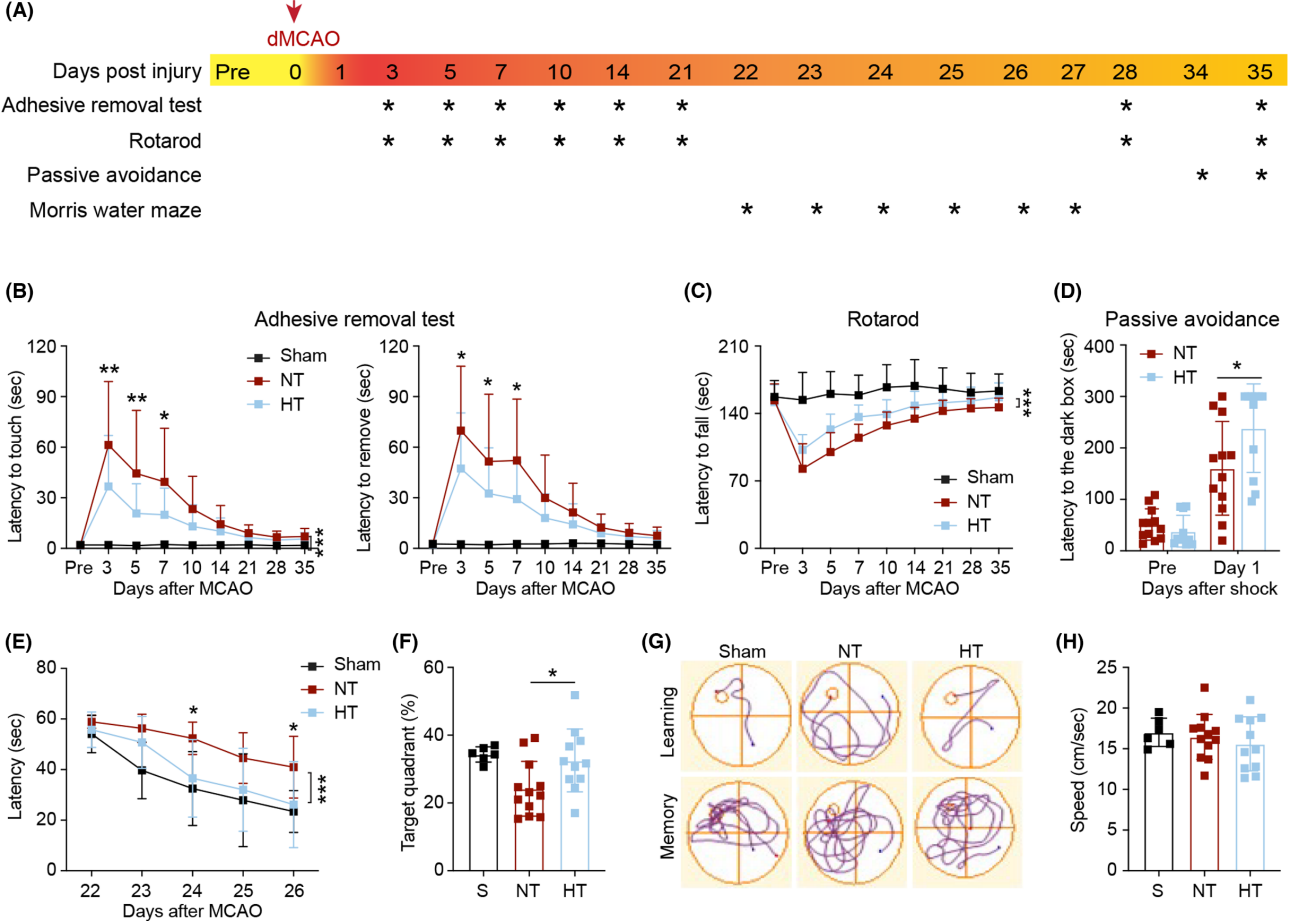
Selective brain hypothermia alleviates long‐term neurological dysfunction in an ischemic stroke model in aged female mice. Mice were subjected to distal MCAO (dMCAO) or sham surgery. Normothermia (NT) or hypothermia (HT) was induced for 50 minutes immediately after surgery. (A) Schematic diagram showing the neurological function test design and procedures. (B, C) Adhesive removal (B) and rotarod (C) tests were used to examine the sensorimotor function of mice after dMCAO. (D) Passive avoidance tests were used to assess the ability of mice to learn and remember after dMCAO. (E–H) Morris water maze test was used to measure the ability of mice to learn and remember after dMCAO. Spatial learning tests were performed 22–26 days after dMCAO or sham surgery (E). Spatial memory tests were conducted 27 days after dMCAO or sham surgery (F). Representative trail graphs (G). Average swimming speed was recorded when performing the memory test (H). Data are shown as mean ± SD. *p < 0.05, **p < 0.01, ***p < 0.001 NT vs. HT. ns, no significance. *n* = 6 for sham, *n* = 12 for NT, and *n* = 11 for HT.

Hypothermia has been shown to improve functional independence in adult male mice with acute ischemic stroke.^19^ To determine whether mild selective brain hypothermia could alleviate neurological deficits in aged female mice, we conducted several neurobehavioral examinations up to 35 days after acute cerebral ischemia (Figure 1A). dMCAO was performed on 18‐month aged female C57BL/C mice. Then, immediately following the dMCAO procedure, mice were treated with mild selective brain hypothermia for 50 min at a temperature of 31–32°C. Although ischemia induced severe sensorimotor defects in both NT and HT mice, during the long‐term recovery period, the HT group showed greater improvements in sensorimotor function compared to the NT group based on the adhesive removal (Figure 1B) and rotarod (Figure 1C) tests. The passive avoidance test was conducted in mice on day 34 (pre‐shock) and day 35 (after shock) post‐ischemia. We found that the latency to enter the dark chamber was longer in HT mice than NT mice (Figure 1D). Spatial learning and memory performance were examined using the Morris water maze test 22–27 days after dMCAO. We found that the escape latency to locate the hidden platform was shorter in HT mice than NT mice (Figure 1E). During the probe test, the HT group spent more time in the target quadrant than the NT mice (Figure 1F). These results indicated that mild selective brain hypothermia promoted passive avoidance learning, spatial learning, and spatial memory after dMCAO in aged female mice.

### 3.2 Mild selective brain hypothermia reduces neuronal loss after acute cerebral ischemia in aged female mice

**FIGURE 2:**
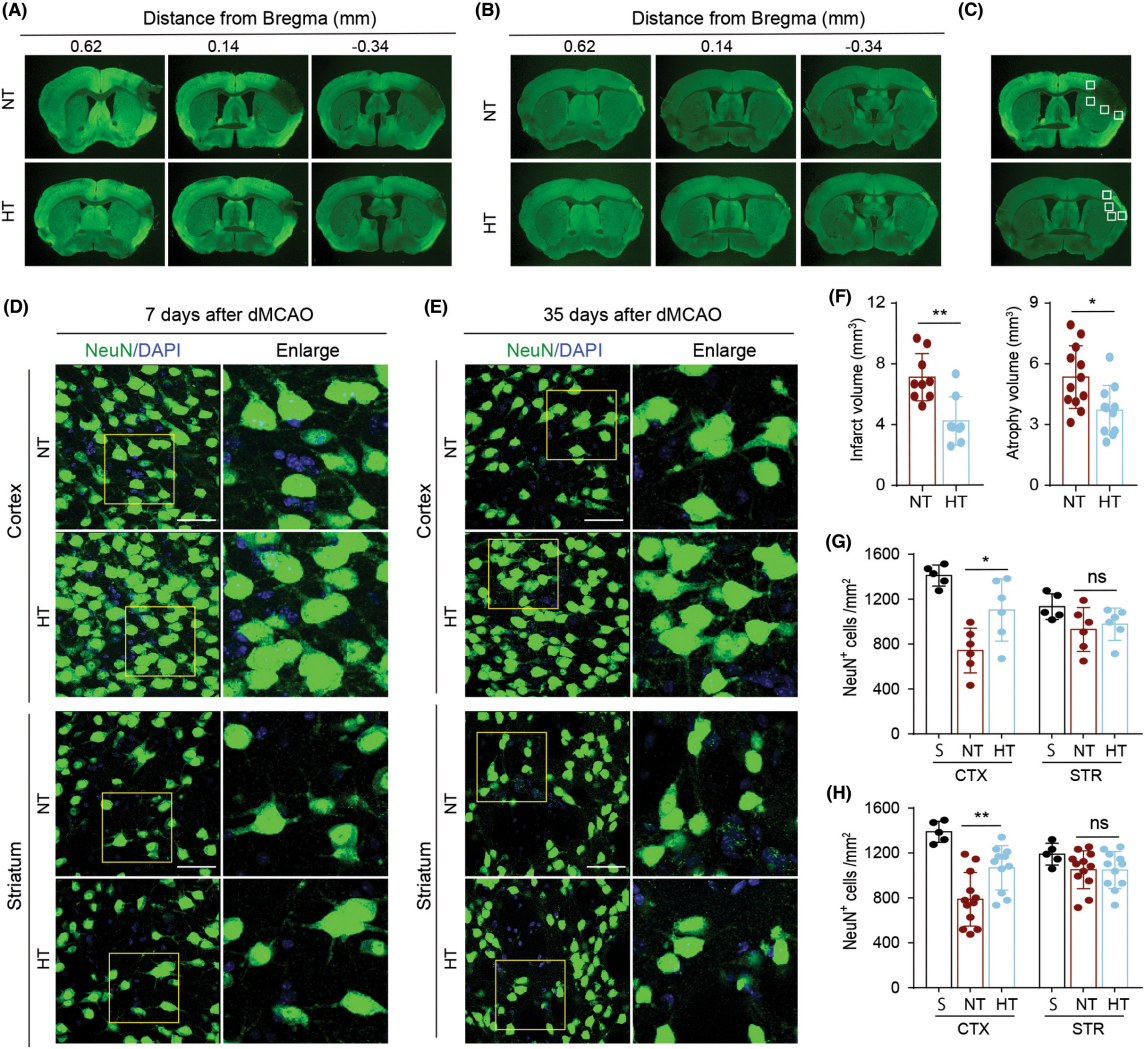
Selective brain hypothermia reduces infarct volume and brain atrophy in an ischemic stroke model in aged female mice. Mice were subjected to distal MCAO (dMCAO) or sham surgery. Normothermia (NT) or hypothermia (HT) was induced for 50 minutes immediately after dMCAO. (A, B) Infarct volume (A) and brain atrophy (B) were assessed in coronal brain sections by immunostaining for MAP2 (green) 7 (A) and 35 days (B) after dMCAO. (C–E) Neuronal loss was evaluated in coronal brain sections by immunostaining for NeuN (green) 7 and 35 days after dMCAO. Boxes show the peri‐infarct cortex (CTX) and striatum (STR) areas where the immunofluorescence images were obtained (C). Representative images of NeuN^+^ cells 7 days after dMCAO (D). Representative images of NeuN^+^ cells 35 days after dMCAO (E). (F) Quantification of infarct volume (left, *n* = 9 for NT and *n* = 8 for HT) and brain atrophy volume (right, *n* = 12 for NT and *n* = 11 for HT). (G) Quantification of NeuN^+^ cells in the CTX and STR areas 7 days after dMCAO, *n* = 5 for sham, *n* = 6 for NT and *n* = 6 for HT. (H) Quantification of NeuN^+^ cells in the CTX and STR areas 35 days after dMCAO, *n* = 5 for sham, *n* = 12 for NT and *n* = 11 for HT. Scale bar: 50 μm. Data are shown as mean ± SD. *p < 0.05, **p < 0.01 NT vs. HT, ns, no significance.

Our behavioral tests demonstrated that mild selective brain hypothermia alleviated neurological dysfunction in aged female ischemic mice. To determine the underlying mechanisms, we examined the preserved gray matter of mice after dMCAO. Using MAP‐2 immunostaining, we found that both the infarct volume at day 7 post‐dMCAO and atrophy volume at day 35 post‐dMCAO was lower in HT mice than NT mice (Figure 2A,B,F). In addition, we calculated the number of NeuN^+^ cells in the peri‐infarct area at days 7 and 35 post‐ischemia. We found that dMCAO induced a loss of NeuN^+^ cells in the peri‐infarct areas of both the cortex (CTX) and striatum (STR). The number of NeuN^+^ cells was higher in HT mice than NT mice at days 7 and 35 after dMCAO (Figure 2D,E,G,H). However, statistically significant differences were only observed in the peri‐infarct areas of the CTX and not the STR (Figure 2G,H). This is presumably because dMCAO tends to induce damage in the CTX rather than the STR. These results suggested that mild selective brain hypothermia elicited both short‐ and long‐term protective effects on the gray matter of aged female ischemic mice.

### 3.3 Mild selective brain hypothermia protects against white matter injury after acute cerebral ischemia in aged female mice

**FIGURE 3:**
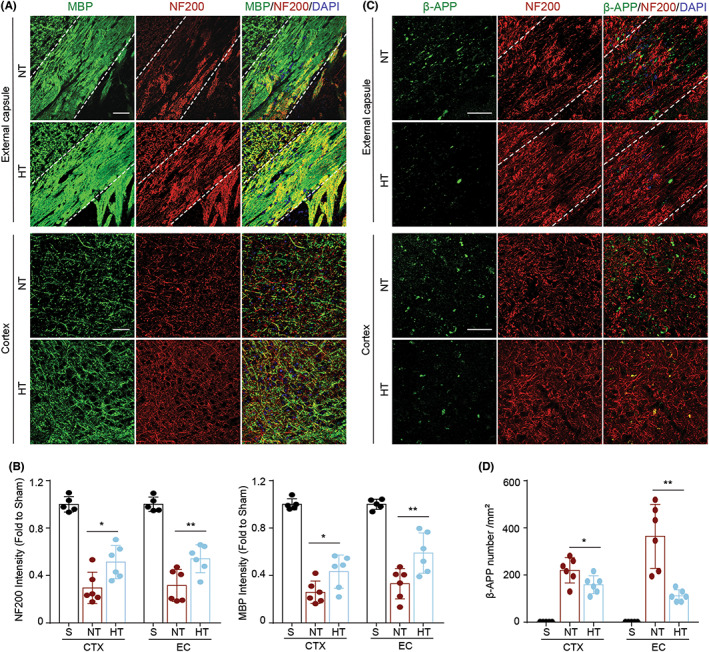
Hypothermia protects the integrity of the white matter in aged female mice 7 days after ischemic stroke. Mice were subjected to distal MCAO (dMCAO) or sham surgery. Normothermia (NT) or hypothermia (HT) was induced for 50 min immediately after dMCAO. Coronal brain sections were stained for myelin basic protein (MBP), NF200 (an axonal marker), and beta‐amyloid precursor protein (β‐APP; a marker of axonal damage). (A) Representative images of double‐labeled MBP/NF200 immunofluorescence staining in the ipsilateral peri‐infarct external capsule (EC) and cortex (CTX). (B) Quantification of NF200 (left) and MBP (right) immunofluorescence intensity. (C) Representative images of double‐labeled β‐APP/NF200 immunofluorescence staining in the ipsilateral peri‐infarct external capsule (EC) and cortex (CTX). (D) Quantification of the number of β‐APP per mm^2^. Scale bar: 50 μm. Data are shown as mean ± SD. *p < 0.05, **p < 0.01 NT vs. HT. *n* = 5 for sham, *n* = 6 for NT and *n* = 6 for HT.

Since white matter has a critical role in neurobehavioral functions, we next examined white matter damage in the external capsule (EC) and peri‐infarct CTX areas of mice at day 7 post‐dMCAO. Immunofluorescence staining of MBP and NF200, markers for myelin and axon, respectively, was used to determine the integrity of the white matter. We found increased expression of NF200 and MBP (Figure 3A,B) in both the EC and peri‐infarct CTX areas of HT mice compared to NT mice, suggesting that selective brain cooling decreased myelin loss after ischemia. In addition to loss of myelin (demyelination), white matter injury also involves axonal damage. Here, we used β‐APP as a robust marker for axonal damage and found that mild selective brain hypothermia reduced β‐APP accumulation (Figure 3C,D) in both the EC and peri‐infarct CTX areas. These results suggested that mild selective brain hypothermia protects against white matter injury 7 days after acute ischemic stroke in aged female mice.

### 3.4 Mild selective brain hypothermia suppresses the harmful immunological response after acute cerebral ischemia injury in aged female mice

**FIGURE 4:**
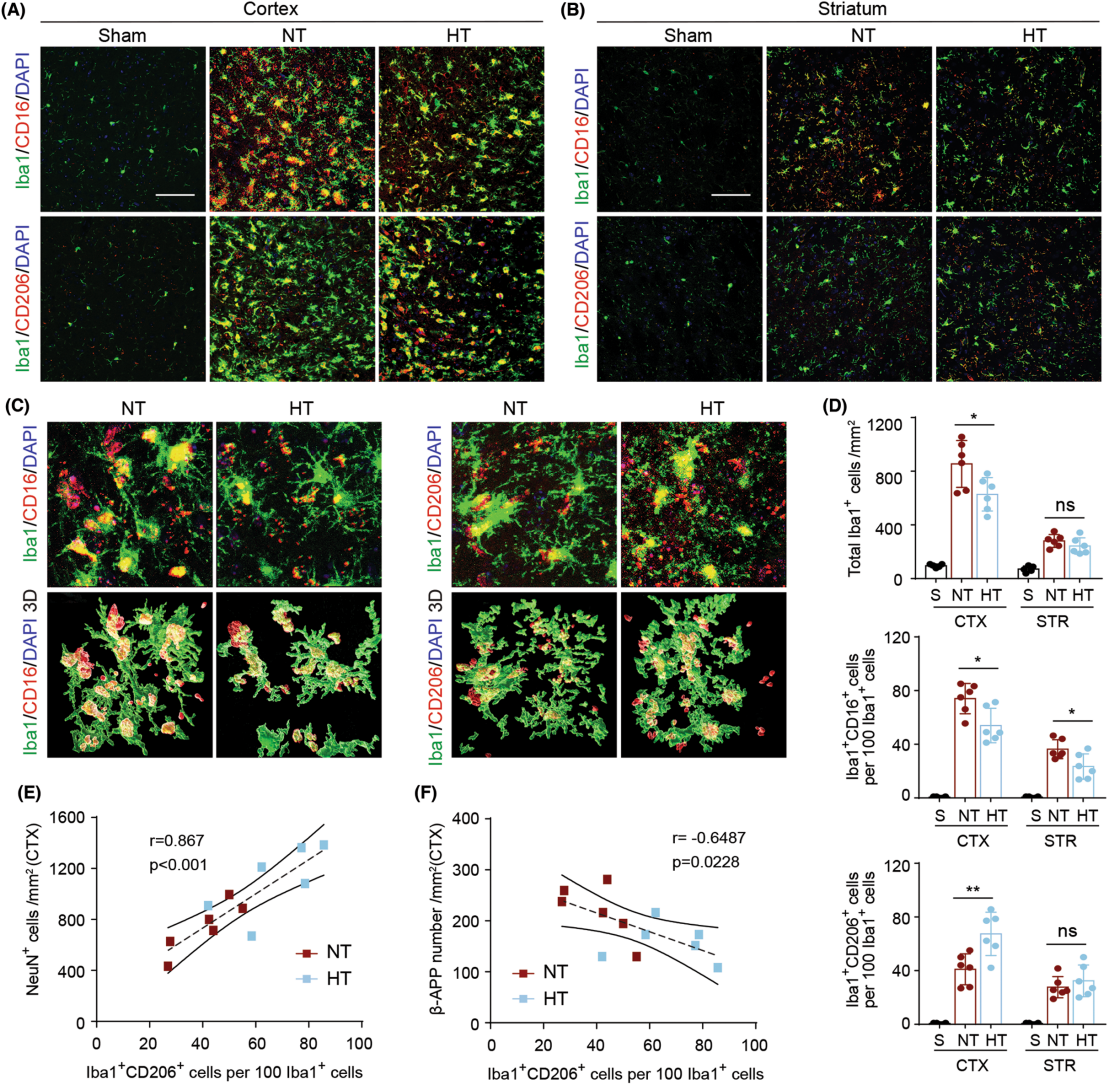
Hypothermia promotes a shift of microglia/macrophages towards an anti‐inflammatory phenotype in aged female mice 7 days after ischemic stroke. Mice were subjected to distal MCAO (dMCAO) or sham surgery. Normothermia (NT) or hypothermia (HT) was induced for 50 min immediately after dMCAO. Coronal brain sections were stained for Iba‐1 (a microglial/macrophage marker) and CD206 (an anti‐inflammatory marker) or CD16 (a pro‐inflammatory marker). (A) Representative images of Iba1/CD16 and Iba1/CD206 immunofluorescence staining in the ipsilateral peri‐infarct cortex (CTX) regions. (B) Representative images showing Iba1/CD16 and Iba1/CD206 immunofluorescence staining in the ipsilateral peri‐infarct striatum (STR) regions. (C) Representative magnified 3‐dimensional images of Iba1/CD16 and Iba1/CD206 staining. (D) Quantification of the total number of Iba1^+^ cells (upper panel), Iba1^+^/CD16^+^ cells (middle panel), and Iba1^+^/CD206^+^ cells (lower panel). The number of double‐positive cells was expressed as the number over 100 Iba1^+^ cells. *n* = 5 for sham (S), *n* = 6 for NT and *n* = 6 for HT. (E) Pearson correlation analysis of NeuN^+^ cells with Iba1^+^/CD206^+^ cells in the CTX region, *n* = 6 per group. (F) Pearson correlation analysis of β‐APP^+^ cells with Iba1^+^/CD206^+^ cells in the CTX region, *n* = 6 per group. Scale bar: 50 μm. Data are shown as mean ± SD. *p < 0.05, **p < 0.01 NT vs. HT, ns, no significance.

Brain‐resident microglia/macrophages have detrimental effects when acute ischemia occurs.^20^, ^21^, ^22^, ^23^ Our previous studies demonstrated that mild selective brain hypothermia promoted a shift from harmful pro‐inflammation microglia to beneficial anti‐inflammation microglia after acute ischemic stroke in adult male mice.^11^, ^12^ In this study, we sought to determine whether hypothermia had a similar effect in aged female mice after ischemic stroke. Immunofluorescence staining of Iba‐1 (a microglia/macrophage marker), CD16 (a pro‐inflammation microglia/macrophage marker), and CD206 (an anti‐inflammation microglia/macrophage marker) was used to examine the activation and phenotype of microglia/macrophages (Figure 4A–C). We found that HT mice had fewer Iba‐1^+^ cells than NT mice in the peri‐infarct CTX areas 7 days post‐dMCAO (Figure 4D, upper panel). Co‐staining of Iba‐1 and CD16 showed that selective brain cooling resulted in a decrease in detrimental pro‐inflammation microglia/macrophages post‐dMCAO (Figure 4D, middle panel). At the same time, co‐staining of Iba‐1 and CD206 demonstrated that hypothermia led to an increase in beneficial anti‐inflammation microglia/macrophages in the peri‐infarct CTX areas (Figure 4D, lower panel). These results demonstrated that selective brain cooling promoted a shift from detrimental pro‐inflammation microglia to beneficial anti‐inflammation microglia in aged female stroke mice. Next, we used Pearson's correlation analysis to examine the correlation between the number of CD206^+^/Iba1^+^ CTX cells and either β‐APP accumulation in the CTX or the number of NeuN^+^ cell in the CTX 7 days after dMCAO. We found that the number of CD206^+^/Iba1^+^ cells was positively correlated with the number of NeuN^+^ cells (Figure 4E), but negatively correlated with β‐APP accumulation (Figure 4F) 7 days after dMCAO. These results suggested that CD206^+^ microglia/macrophages might be involved in the induction of gray and white matter protection by mild selective brain hypothermia after ischemic brain injury in aged female mice.

### 3.5 Mild selective brain hypothermia has long‐term protective effects against white matter injury after acute cerebral ischemia in aged female mice

**FIGURE 5:**
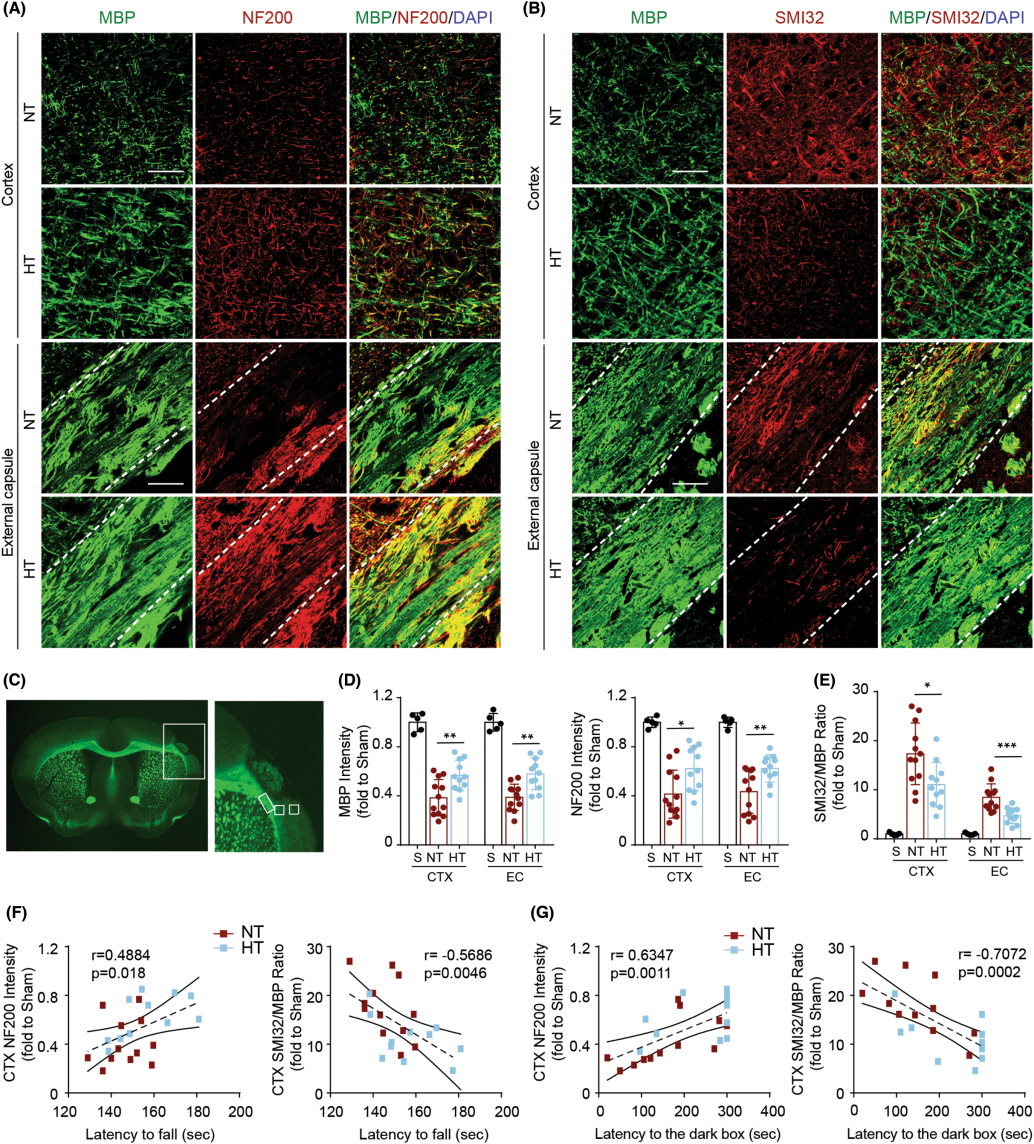
Hypothermia improves the long‐term integrity of the white matter in aged female mice 35 days after ischemic stroke. Mice were subjected to distal MCAO (dMCAO) or sham surgery. Normothermia (NT) or hypothermia (HT) was induced for 50 min immediately after dMCAO. Coronal brain sections were stained for myelin basic protein (MBP), SMI‐32 (a demyelination marker), and NF200 (an axonal marker). (A, B) Representative images showing double‐labeled MBP/NF200 (A) and MBP/SMI32 (B) immunofluorescence staining in the ipsilateral peri‐infarct cortex (CTX) and external capsule (EC) regions. (C) Boxes showing the peri‐infarct CTX and EC areas where the immunofluorescence images were obtained. (D) Quantification of MBP (left) and NF200 (right) immunofluorescence intensity. (E) Quantification of the ratio of SMI‐32 to MBP (SMI32/MBP) immunofluorescence intensity. (F) Correlation of NF200 staining intensity in the CTX with latency to fall in the rotarod test (left) and SMI32/MBP staining intensity in the CTX with latency to fall in the rotarod test (right). (G) Correlation of NF200 staining intensity in the CTX with latency to enter dark chamber in passive avoidance test (left) and SMI32/MBP staining intensity in the CTX with latency to enter dark chamber in passive avoidance test (right). Scale bar: 50 μm. Data are shown as mean ± SD. *p < 0.05, **p < 0.01, ***p < 0.001 NT vs. HT, *n* = 5 for sham, *n* = 12 for NT and *n* = 11 for HT.

Having demonstrated that mild selective brain hypothermia led to a decrease in neuronal loss and protected against white matter damage in mice 7 days post‐dMCAO, we next examined whether hypothermia had long‐term protective effects on the white matter 35 days post‐dMCAO. The ratio of demyelinated to myelinated axons was determined by immunofluorescence staining with SMI‐32, a marker for demyelinated axons, and MBP (Figure 5A,B). We found that hypothermia increased MBP and NF200 expression (Figure 5D) in both the EC and peri‐infarct CTX areas. The increase in the SMI‐32/MBP ratio was reversed in both the EC and CTX areas of HT mice at 35 days post‐dMCAO (Figure 5E). Furthermore, NF200 expression was positively correlated with the latency to fall, while the SMI‐32/MBP ratio was negatively correlated with the latency to fall in the rotarod test at 35 days post‐dMCAO (Figure 5F). In addition, we found that NF200 expression was positively correlated with the latency to enter the dark chamber, while the SMI‐32/MBP ratio was negatively correlated with the latency to enter the dark chamber in the passive avoidance test at 35 days post‐dMCAO (Figure 5G). These results suggested that mild selective brain hypothermia has long‐term protective effects against white matter injury after acute cerebral ischemia in aged female mice.

## 4 DISCUSSION

In this study, we demonstrate the neuroprotective effects of mild selective brain hypothermia in an ischemic model of aged female mice. We show that hypothermic therapy improves short‐term and long‐term neurological function in mice by alleviating gray and white matter injury and suppressing harmful inflammatory responses.

Although thrombectomy has been used to treat large‐vessel occlusion stroke in recent years, one‐third of patients who receive successful vessel recanalization still experience poor clinical outcomes.^24^ Of the many neuroprotective therapies that have been studied in the past few decades, hypothermia has proved to be one of the most promising strategies, due to its multifaceted neuroprotective effects.^25^, ^26^, ^27^ However, the majority of studies on preclinical hypothermia have only focused on male adult animals. Since ischemic stroke affects the aged population, in both males and females, these male adult animal models are not a true representation of the actual affected population. In this study, we used aged female mice to confirm the protective effect of mild selective brain hypothermia on focal ischemia brain injury induced by permanent dMCAO. We found that selective brain hypothermia reduced the infarct volume and neuronal loss in our aged female mice ischemic stroke model. Furthermore, neurobehavioral tests demonstrated that selective brain hypothermia improved sensorimotor and memory functions in aged female ischemic stroke mice. Our results indicated that selective brain hypothermia has neuroprotective effects in aged female ischemic stroke mice, as well as in male mice.

In addition to selective brain hypothermia, systemic hypothermia has also been used as a means of therapeutic hypothermia. Systemic cooling can be induced by physical or pharmacological methods.^28^ Furthermore, pharmacological cooling may have more robust neuroprotective effects through additional mechanisms.^28^ Thus, combining physical and pharmacological hypothermia may achieve the same or even more enhanced neuroprotective effects.^29^ In contrast, whole‐body hypothermia has been associated with several adverse effects, such as immunodepression, pneumonia, cardiovascular deficiency, coagulopathy, and severe shivering.^26^ In this study, we used selective brain hypothermia to avoid decreasing the core body temperature and increasing the number of potential systemic complications. The present data showed that selective brain hypothermia led to a decrease in neuronal loss, improved behavioral outcomes, induced the polarization of microglia/macrophages towards an anti‐inflammatory phenotype and reduced white matter damage after ischemic stroke in aged female mice.

White matter integrity is essential to sensorimotor and cognitive function.^30^, ^31^ White matter damage, including both myelin loss and axonal degeneration, can induce signal transmission abnormalities between different brain areas, which leads to neurobehavioral deficits.^32^ White matter injury is inevitable in ischemic stroke patients.^33^ However, the effects of selective brain hypothermia on white matter damage have rarely been studied in aged female ischemic stroke mice. Consistent with a previous study,^34^ we observed serious myelin and axonal injury in female ischemic mice. These injuries to the white matter were reduced at both days 7 and 35 post‐dMCAO by the administration of selective brain hypothermia. Furthermore, we found that myelin integrity was positively correlated with the long‐term neurological function of aged female mice after dMCAO. Thus, we believe that the protection or restoration of white matter integrity by selective brain cooling may improve sensorimotor and cognitive functions in aged female ischemic stroke mice.

Many studies have demonstrated that hypothermia leads to a decrease in the activation of resident microglia and promotes a shift of microglia/macrophages towards an anti‐inflammatory phenotype.^35^, ^36^, ^37^ Previous studies have also shown that hypothermic therapy increased the number of CD206^+^ microglia/macrophages, which are associated with white matter protection after ischemic stroke.^37^, ^38^, ^39^ Furthermore, anti‐inflammation microglia have been shown to drive oligodendrocyte differentiation and improve remyelination,^32^ while pro‐inflammation microglia can aggravate oligodendrocyte cell death and white matter damage after brain injury.^40^, ^41^ Different microglial phenotypes are not only associated with white matter damage but also affect the survival of the gray matter after brain injury. Hu et al demonstrated that anti‐inflammatory CD206^+^ microglia/macrophages are beneficial to the survival of neurons after ischemic stroke.^21^ Consistent with these studies, we found that selective brain hypothermia‐induced protection of the white and gray matter after dMCAO was also associated with an increase in anti‐inflammatory microglia/macrophages. Thus, our findings demonstrated that hypothermic therapy is beneficial to aged female mice by suppressing the immune response.

In summary, this study demonstrated that selective brain hypothermia improves sensorimotor and cognitive functions in an aged female mouse model of ischemia. This functional protection may be due to its promotion of white and gray matter integrity. In addition, we found that selective brain hypothermia suppressed activation of microglia and promoted polarization of microglia to the beneficial anti‐inflammation phenotype after ischemic stroke in aged female mice. This anti‐inflammation phenotype is positively associated with neuronal survival and negatively associated with white matter injury. Our findings may partially describe the underlying mechanisms of neuroprotection against acute cerebral ischemic stroke in aged female mice. Some previous studies used adult male rats^42^, ^43^ or 13‐ to 14‐week‐old male spontaneously hypertensive rats^44^ to investigate the effects of hypothermia on permanent ischemia. Their results showed that hypothermia had no positive effect on infarct volume reduction in the permanent MCAO model. This is not consistent with our study. Maybe, it is due to the difference of gender, age, or species of the experimental animal. Further studies are warranted to explore the exact protective mechanisms of selective brain hypothermia in an aged female mouse model of ischemia. More importantly, further experiments are required to understand the mechanistic differences between female and male animal ischemic models.

## AUTHOR CONTRIBUTIONS

Liqiang Liu contributed to the study concept and design, acquisition, statistical analysis, interpretation of data, and drafting and revising the manuscript. Jia Liu contributed to the study design, acquisition, and interpretation of data, and revising the manuscript. Junxuan Lyu, Ming Li, Wei Su, and Shejun Feng contributed to the acquisition and interpretation of data and revising the manuscript. Xunming Ji contributed to the study concept, design, supervision, statistical analysis, interpretation of data, and drafting and revising the manuscript.

## CONFLICT OF INTEREST

Dr. Jia Liu is an Editorial Board member of CNS Neuroscience and Therapeutics and a co‐author of this article. To minimize bias, she was excluded from all editorial decision‐making related to the acceptance of this article for publication. Other authors declare that they have no conflicts of interest.

## Data Availability

The data that support the findings of this study are available from the corresponding author upon reasonable request.
